# Myocardial Bridging as a Differential Diagnosis for Myocardial Infarction: A Case Report

**DOI:** 10.7759/cureus.37200

**Published:** 2023-04-06

**Authors:** Carter Gay, Colby Kihara, Arsh N Patel, Katie Oakley, Laurence Stolzenberg, Allen Schmidt

**Affiliations:** 1 Medicine, Alabama College of Osteopathic Medicine, Dothan, USA; 2 Orthopedic Surgery, Alabama College of Osteopathic Medicine, Dothan, USA; 3 Internal Medicine, Decatur Morgan Hospital, Decatur, USA

**Keywords:** coronary angiography, st-segment elevation, myocardial infarction, myocardial ischemia, myocardial bridging

## Abstract

We present a case of a 55-year-old male who presented to the emergency department with complaints of acute onset of chest pain. As part of the workup, an electrocardiogram (ECG) revealed ST-segment elevation in leads V1-V5. Upon cardiac catheterization, myocardial bridging of the left anterior descending (LAD) artery was discovered without evidence of any obstructive coronary artery disease.

The purpose of this report is to add to the existing literature that myocardial bridging, although traditionally thought to be a benign pathology, can also present risks for ischemia and infarction.

## Introduction

Myocardial bridging is an anomaly of the coronary arteries, which course along the wall of the epicardium before branching off to supply cardiac tissue. In cases of myocardial bridging, the epicardial coronary artery travels through the myocardium for a portion of its length, instead of lying superficially on the cardiac wall [1]. This may be problematic as the vessel is susceptible to compression during myocardial contraction. While generally considered to be a benign pathology, there is increasing evidence to support the risk of ischemia, infarction, arrhythmia, syncope, and sudden cardiac death that stems from myocardial bridging [2]. Here, we present a case of myocardial ischemia that developed during strenuous exercise in a patient with a previously unknown myocardial bridge anomaly.

This article was previously presented as a poster abstract at the 2022 Alabama College of Osteopathic Medicine (ACOM)/Society of Hospital Medicine Wiregrass Chapter Poster Day on November 29, 2022.

## Case presentation

**Figure 1:**
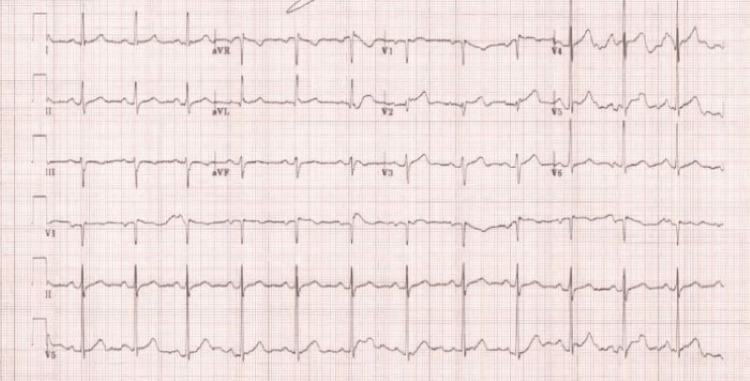
EKG indicating ST-segment elevation in leads V1-V5. EKG, electrocardiogram

A 55-year-old male presented to the emergency department with a sudden onset of severe chest pain of 40 minutes duration. The symptoms first began while the patient was exercising and was not relieved with rest. The pain was localized predominantly to the left side of the chest, did not radiate, and was described as a heavy, pressure-like feeling. The patient had experienced some chest discomfort before this episode but attributed it to dyspepsia, as his past medical history included a Helicobacter pylori infection. The patient’s current medications included omeprazole 40 mg daily, ampicillin 500 mg three times a day, and levofloxacin 500 mg daily. The patient denied any history of tobacco, alcohol, or illicit drug use. All of his vital signs were within normal limits apart from his blood pressure and mean arterial pressure, which read 142/108 and 119.33 mmHg, respectively. A physical exam revealed clear lungs with equal breath sounds bilaterally. Heart rate and rhythm were regular with no discernable murmurs, gallops, or rubs. Radial and pedal pulses were both 3+ bilaterally. There were no signs of peripheral edema, clubbing, or cyanosis. Bloodwork included the following: a complete blood count (CBC), basic metabolic panel (BMP), D-dimer, pro-brain natriuretic peptide (pro-BNP), prothrombin (PT), partial thromboplastin time (PTT), and a troponin test, all of which revealed no abnormalities. ECG revealed ST-segment elevation in leads V1-V5 (Figure 1).

**Figure 2:**
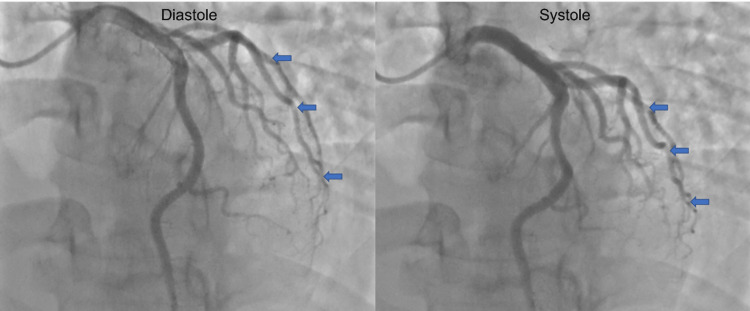
Coronary angiography revealed compression of coronary vessel segments during systole, as compared to diastole.

The patient was started on anticoagulation therapy but required transport by helicopter to another facility with cardiac catheterization abilities. However, cardiac catheterization found no evidence of obstructive coronary artery disease. Instead, myocardial bridging of the left anterior descending (LAD) artery was discovered and determined to be the source of the myocardial ischemia (Figure 2). In a review of the Schwarz classification system for myocardial bridging and treatment, there was evidence of ischemia present on the stress test, which falls under Schwarz type B [3]. For management, the patient was started on diltiazem 120 mg daily to provide ventricular rate control and improve coronary vasodilation. The patient was discharged after 24 hours in stable condition. Follow-up at two weeks and one month revealed no recurrence of symptoms or limitation with daily activities.

## Discussion

Myocardial bridging is a congenital anomaly that may be associated with several possible complications; however, its presence is still questioned in the literature for clinical relevance [4]. The anatomical presence of myocardial bridging occurs when a segment of the epicardial coronary artery runs through the myocardium. The concern occurs during systole when the coronary artery becomes compressed, which may cause symptoms of angina, arrhythmia, depressed left ventricular function, myocardial stunning, and cardiac death. The true prevalence in the population for myocardial bridging is not clearly defined; however, one study examined 118 patients via coronary computed tomographic angiography (CCTA) and found the prevalence to be approximately 30%, which is in concordance with the rates determined by most autopsy report studies [5].

As most patients are asymptomatic throughout life, diagnostic approaches pose a challenge as true diagnostic accuracy measures have not been thoroughly studied [6]. The two standards for diagnosis include noninvasive and invasive methods. The noninvasive methods are cardiac computer tomography angiography and stress echocardiography. The invasive and most common diagnostic methodology is the use of coronary angiography, which will demonstrate the systolic compression of vessel segments and the characteristic *milking effect,* which is the decompression of vessels during diastole. Intravascular ultrasound, intracoronary Doppler, and assessment of fractional flow reserve are newer techniques that may allow for a fuller understanding of the pathologic effects that bridging may have on hemodynamics and flow [7].

The management for myocardial bridging has been broken down based on the Schwarz classification type and is displayed in Table 1 [3]. Overall, symptomatic treatment has focused more on pharmacologic therapy rather than surgical. β-blockers and calcium channel blockers (CCBs) are considered to be first-line treatment options [7]. Both β-blockers and CCBs decrease heart rate, which improves diastolic filling time and decreases the compression of the bridged vessel segments. CCBs also have the advantage of coronary vasodilatory properties, which can further reduce the compression of bridged segments and relieve vasospasm. Nitrates are considered contraindicated because nitroglycerin has been shown to accentuate the systolic compression of bridged segments by decreasing preload and vasodilating the adjacent nonbridged coronary vessels [3].

**Table 1:** Schwarz classification for myocardial bridging. QCA, quantitative coronary angiography; CFR, coronary flow reserve; BB, β-blocker; CCB, calcium channel blocker

Schwarz classification	Criteria	Ischemia	Management
Type A	Incidental angiographic finding	-	Reassurance
Type B	Ischemia on stress test	+	BB or CCB
Type C	Altered intracoronary hemodynamics (based on QCA/CFR/Doppler)	+/-	BB or CCB +/- Revascularization

A newly developed technique, surgical unroofing, is a potential option for refractory cases or as a last resort, but the procedure is not without risk. Surgical unroofing involves cutting into the myocardium to expose the tunneled vessels, which should resolve the ischemia but may also damage the healthy heart muscle and impair cardiac conductivity [8].

## Conclusions

Myocardial bridging, once believed to be a benign pathology, can increase the risk for ischemia and infarction. Despite being an anatomical variation, the high degree of variability in the location and severity of the bridging makes it difficult to manage. This case highlights the importance of this condition as an alternative diagnosis in cases of ST-elevation myocardial infarction (STEMI) with negative cardiac biomarkers.
